# Cutaneous Angiosarcoma of the Foot: A Case Report and Review of the Literature

**DOI:** 10.1155/2014/657876

**Published:** 2014-12-09

**Authors:** Sharang Tenjarla, Lucy Ashley Sheils, Theresa M. Kwiatkowski, Sheema Chawla

**Affiliations:** ^1^Department of Radiation Oncology, Rochester General Hospital, 1425 Portland Avenue, Rochester, NY 14621, USA; ^2^Department of Pathology, Rochester General Hospital, 1425 Portland Avenue, Rochester, NY 14621, USA

## Abstract

Primary Angiosarcoma of the skin of the foot is very rare. Angiosarcoma is typically treated with resection and wide-field postoperative radiation therapy. Chemotherapy and radiation therapy have also been used. Regardless of the treatment, the risk of local and distant relapse remains high for this disease. We present a case of an elderly patient who developed cutaneous angiosarcoma of the foot. It posed as a diagnostic dilemma at presentation. Chronic lymphedema was a possible predisposing factor. Given his age, preexisting renal dysfunction, refusal of surgery, and preference not to receive chemotherapy, the patient was ultimately treated with definitive radiotherapy. We present this case because of its rare site, unique presentation and delay in diagnosis of the condition, and attainment of an excellent response to radiation at the time of follow-up. We also review the current literature on this topic.

## 1. Introduction

Angiosarcoma is a rare, malignant neoplasm comprising 1–3% of adult soft tissue sarcomas [1–4]. This is typically a tumor of older individuals with a median age of 70–75 years and a male predominance, having a predilection for the scalp and central area of the face. Cutaneous angiosarcoma is clinically aggressive. The reported 5-year survival rate ranges from 12 to 24% [1, 5]. The neoplasm tends to invade tissue more widely than is clinically apparent and is thus prone to incomplete excision. Majority of patients present with locally advanced disease, regional lymph node involvement, or distant metastases at the time of initial diagnosis, all of which are associated with a poor prognosis [4]. The present study describes a case of cutaneous angiosarcoma of the foot in the setting of chronic lymphedema which was treated with definitive radiation alone. The patient was informed that data from the case would be submitted for publication and he provided the required consent.

## 2. Case Presentation

**Figure 1:**
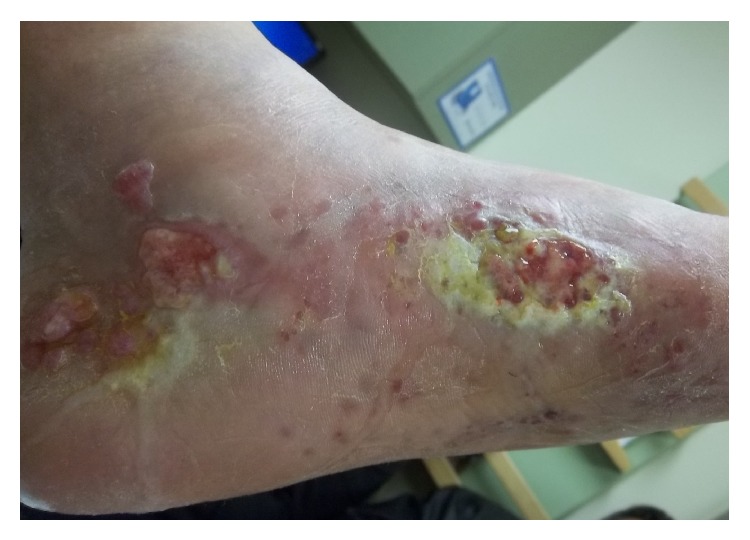
Pretreatment: 5 × 5 cm ulcer on the medial left foot emanating a serosanginous discharge and similar ulcer present posteriorly along with blistering satellite lesions on the plantar surface of the foot.

A 90-year-old Caucasian gentleman with a past medical history of prostatectomy 20 years ago for prostate cancer, chronic venous insufficiency, and lymphedema since a few years presented to the dermatology office with a nonhealing wound in the left medial foot since a few months. He was initially diagnosed with a fungal infection and was given a 4-week course of antifungal agent and wound dressings, not yielding any response to treatment. Bacterial cultures performed a month later showed mixed infection with aerobic and anaerobic flora. He was then given a course of oral and topical antibiotics for 4 weeks, bearing a minimal response to treatment. In the last 2 months before presentation to the clinic, the lesion progressed. On examination, the epicenter of the lesion was located in the medial aspect of the foot. There were two major areas of ulceration in medial foot measuring approximately 5 × 5 cm that emanated a serosanguinous discharge (Figure 1), with blistering satellite lesions in the medial and lateral aspect of the foot. Smaller lesions were also present on the plantar surface of foot. There was nonpitting edema in both extremities. Marked restriction in the movement of the left ankle was noted.

**Figure 2:**
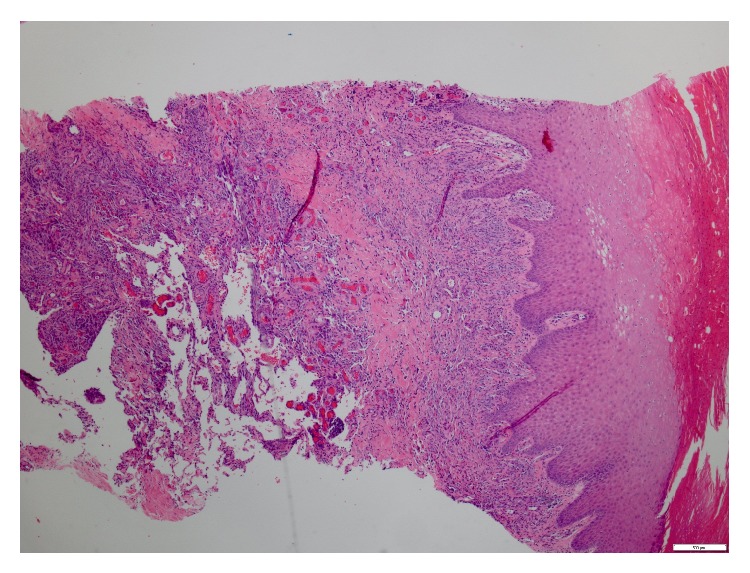
Low power view showing extensive spindle cell proliferation involving dermis, subcutis, and deeper fibroadipose tissue.

**Figure 3:**
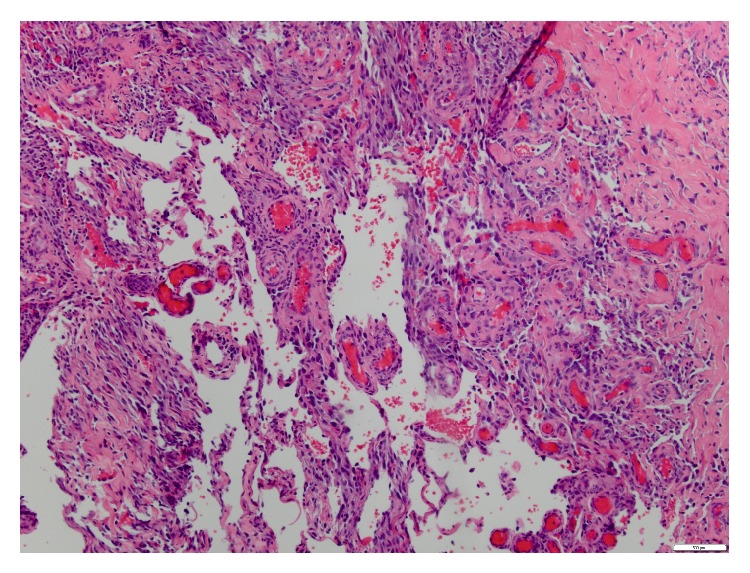
High power view showing multiple vascular sinuses lined by tufts of neoplastic endothelial cells.

**Figure 4:**
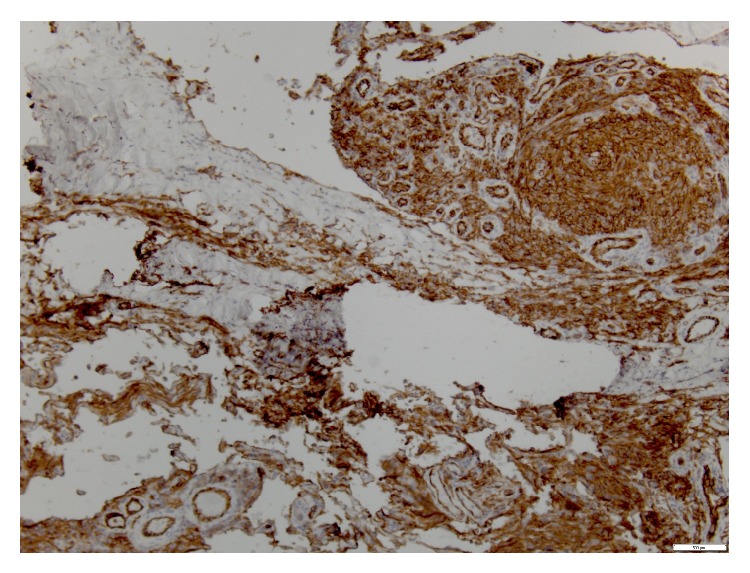
Positivity for vascular marker CD-31.

Subsequently, a punch biopsy of the one of the ulcers revealed cutaneous angiosarcoma, moderate to high grade in differentiation. Microscopically, there was extensive spindle cell proliferation involving dermis, subcutis, and deeper fibroadipose tissue (Figure 2). There were multiple vascular sinuses lined by tufts of neoplastic endothelial cells (Figure 3). Immunohistochemical stains revealed negative staining for cytokeratin while vascular markers CD31 (Figure 4), CD34, and D2-40 were strongly positive. No necrosis was identified in the specimen. Mitotic rate was 0 to 9 mitoses per 10 high power fields. Lymphovascular invasion was indeterminate. 

**Figure 5:**
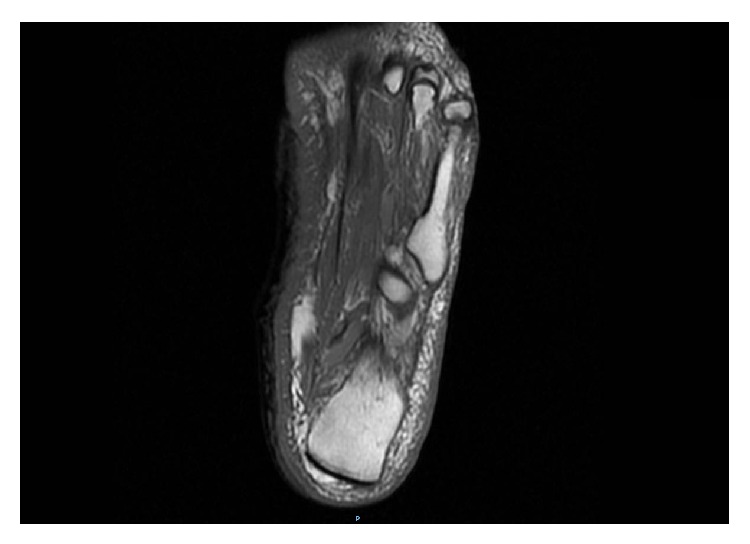
MRI of the left foot with contrast showing diffuse soft tissue T1 hypointense signal within both medial and lateral subcutaneous tissues, which is more prominent in the fat anterior to the Achilles tendon.

**Figure 6:**
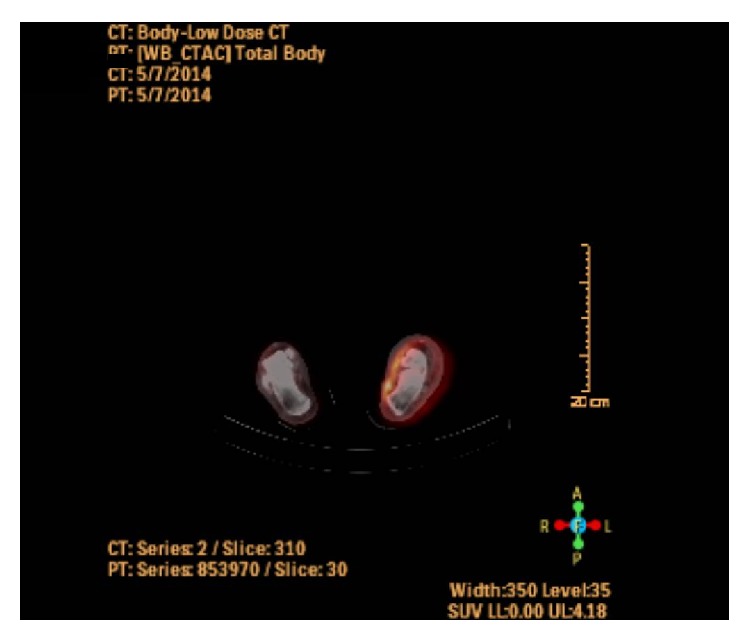
FDG-PET and CT scan showing heterogeneous uptake in the medial and lateral foot with a more focal uptake in the medial foot, anterior to the Achilles tendon.

The patient was referred to the radiation oncology center with the above clinical and pathological data. MRI of the left foot with contrast revealed diffuse soft tissue T1 hypointense (Figure 5) and T2 hyperintense signal within both medial and lateral subcutaneous tissues. This was more prominent in the fat anterior to the Achilles tendon. There was no evidence of invasion in the tendon or the bone. Flourodeoxyglucose (FDG) PET and CT scan revealed heterogeneous uptake in the medial and lateral foot with a more focal uptake in the medial foot, anterior to the Achilles tendon (Figure 6). Two subcentimeter lymph nodes, one in the popliteal region and the other in left groin, showed minimum FDG labelling and were thought to be reactive in nature. No abnormal uptake was seen throughout the body to suggest distant metastases.

Given the diffuse dermal involvement of the foot, the patient was not considered a candidate for upfront surgery. He declined surgical evaluation after preoperative radiation. He was not considered a candidate for chemotherapy because of comorbid conditions, poor renal functions, old age, and reluctance to pursue systemic therapy. He was planned for radiation therapy to both medial and lateral aspects of the foot in addition to the plantar surface using a custom immobilization device. This was done with a combination of photons of 6 MV and electrons of 9 Mev energy to achieve a homogenous dose distribution. A custom bolus was used to build up the radiation dose to the surface. The dose prescribed was 50.4 Gy in 1.8 Gy per fraction to the medial and lateral aspect in 5.5 weeks. The plantar surface of the foot was irradiated to a dose of 30.6 Gy at 1.8 Gy per fraction in 3.5 weeks. He was assessed clinically each week.

**Figure 7:**
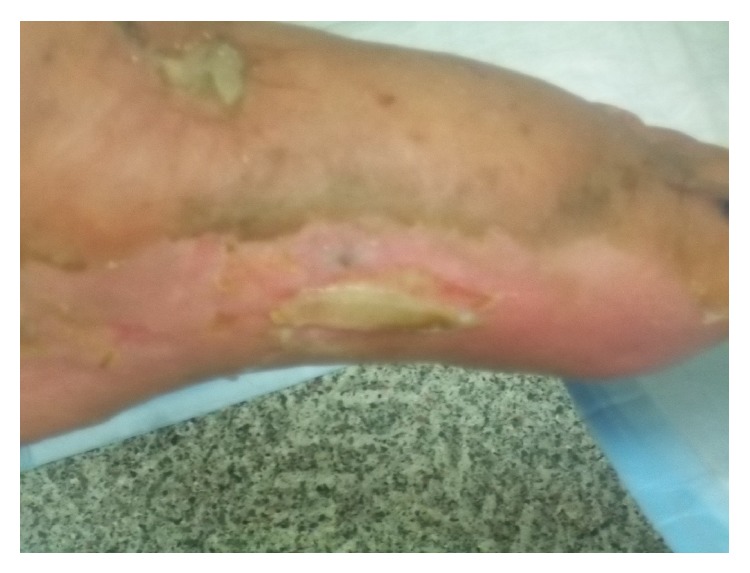
Two months postradiation: marked diminishment of cutaneous ulceration with decline in discharge and drying of satellite nodules.

At the end of the course of radiation, there was anticipated radiation related moist desquamation of the radiated skin which was managed by the wound care center. There was good subjective and objective response to radiation with decline in discharge and excellent diminishment of cutaneous ulceration one month after radiation. At the end of two-month follow-up there was almost complete response and drying of the cutaneous ulceration and satellite nodules in his foot (Figure 7).

## 3. Discussion

Angiosarcoma is a rare and aggressive malignant tumor of vascular endothelial origin. Among all cases of angiosarcoma, one-third occur in the skin, one-fourth in soft tissue, and the remainder in other sites [6]. Radiation therapy, especially for breast cancer, is a predisposing factor. Vascular insufficiency and chronic lymphedema are other predisposing factors in addition to trauma and sun exposure [7]. In many cases, however, the exact cause is unknown [8]. In our case, advanced and chronic venous insufficiency leading to vascular stasis and lymphedema was perhaps the predisposing factor.

As regards clinical appearance, the appearance of cutaneous angiosarcoma can be variable [4] and it can manifest as bruise-like lesions [8], raised purplish-red papules [9], and rosacea-like lesions [10]. Due to the variability in the appearance of cutaneous angiosarcoma, the correct diagnosis can often be delayed. Differential diagnoses include, but are not limited to, eczema [4], Kaposi sarcoma [11], scarring alopecia [12], sebaceous cysts [13], and amelanotic melanoma [14]. Our case is unique that it presented with cutaneous ulceration.

Majority of the patients are noted to be elderly males [15, 16] with Caucasians or fairer skinned people being more commonly affected than darker or black race [17, 18]. More than 90% of the cutaneous lesions are located in the head and neck region. Other non-cutaneous regions are breast and liver. It arises infrequently in the lower extremity [7]. Our case presented in the skin of foot which is very rare although angiosarcoma arising in bones of the foot or femoral artery have been described in the literature [19, 20].

The most common histological patterns include atypical and pleomorphic (rounded, polygonal, or fusiform) endothelial cells exhibiting a diffuse epithelioid or spindle cell proliferation [4, 9, 13, 15]. Immunohistochemical markers include Von Willebrand factor, CD34, CD31, Ulex europaeus agglutinin 1, vascular endothelial growth factor (VEGF), and factor VIII antigen [4, 8]. Our case showed spindle cell proliferation with interspersed vascular sinuses and positivity for CD31 and CD34.

Histological grade [1, 21] and tumor size are important prognostic factors with tumors > 10 cm portending a poor prognosis and tumors < 5 cm correlated with better outcomes [13, 22–24]. High mitotic counts are associated with worse outcomes [25]. In a case series, lymphocytic infiltrate was associated with a good prognosis [26]. Age, sex, and clinical appearance have no prognostic significance [5, 7]. Presence of metastasis, local recurrence, and positive surgical margins correlate with poor outcome [15]. Multifocal disease and depth of invasion (>3 mm) are other poor prognostic features [27]. Local recurrences have been observed in 35% to 86% of cases [16, 28]. Prognosis of angiosarcoma is poor with a reported 5-year survival rate ranging from 12 to 24% [5, 15, 21]. In a series of 48 patients with cutaneous angiosarcoma, 45 patients (94%) had disease recurrences [16]. In the same series, 37 of those patients had distant metastases to the lungs and a median survival time of 4 months. Lung metastases as a common site of spread have also been reported in other series [29, 30]. Other rare reported sites of spread were cardiac and/or vascular metastases [2, 15]. Mendenhall et al. reported a 5-year locoregional control of 40 to 50%, 5-year distant metastasis-free survival of 20 to 40%, and 5-year overall survival of 10 to 30% [31].

Treatment of cutaneous angiosarcoma is based on retrospective data because of the rarity of this disease. Complete resection of the disease is recommended whenever possible, since this disease has a high propensity to recur locally. Surgical excision may not be a feasible option since resectable cutaneous angiosarcoma lesions constitute only a fraction of all cases [4]. Recent studies of primary tumors have reported success with a combined-modality approach of surgical resection followed by postoperative radiation therapy [1, 4, 23, 32]. A retrospective study reported on survival outcomes of 48 patients who were treated for angiosarcoma of face and scalp with either a single modality or a combination of surgery, radiotherapy, chemotherapy, and immunotherapy [16]. The median follow-up for all 48 patients was 13.7 months. Patients who underwent both surgery and radiotherapy (2-year overall survival: 45.8%) had a significantly more favorable overall survival (*P* < 0.0001) compared with patients treated with either surgery or radiotherapy (2-year overall survival: 11.1%) alone and patients who received no surgery or radiotherapy (2-year overall survival: 0%).

Although the combined modality therapy is associated with a better outcome, patients are still at risk for the development of distant metastases [24]. Radiotherapy is a reasonable approach in unresectable or metastatic cases. Care must be taken to achieve full dose to the lesion and to use wide margins due to the diffuse nature of the tumor [33, 34]. It appears to improve local control and possibly overall survival based on the retrospective series in the literature [4, 16, 28, 35], however radiation employed as a single modality of treatment rarely results in complete remission [36, 37]. Radiation doses of > 50 Gy are usually recommended [4] but because of the poor tolerance of hands and feet to radiation [38, 39] we kept our radiation dose to about 50 Gy. Data on the role of chemotherapy in the definitive treatment of cutaneous angiosarcoma is limited and varied. Doxorubicin and Taxanes have been used for treatment in unresectable and metastatic setting [3, 4, 40].

Promising results with bevacizumab [41], sunitinib [42], and sorafenib [43] have also been reported, and their efficacy may be linked to VEGF production in most cases of angiosarcoma. Although single agent therapy with these agents is tolerable, toxicity is significant and patients with advanced age and comorbidities may not qualify for therapy [4]. Photodynamic therapy has been tried by Thong et al. for primary cutaneous angiosarcoma and tumor eradication was achieved with spontaneous remission of neighboring and distant untreated lesions [44].

Since surgery for diffuse involvement of the foot would have resulted in significant morbidity and poor functional outcome, our patient was not considered a candidate for surgery. He refused surgical evaluation after radiation. Systemic therapy and radiotherapy were the next available options. However, he did not want to consider chemotherapy and in any event it he was not a good candidate for aggressive therapy. Therefore, the patient underwent successful treatment with radiotherapy alone as a single modality.

## 4. Conclusion

A rare case of cutaneous angiosarcoma of the foot has been described in this case report. This case portrayed a clinical picture of a nonhealing ulcer with superadded infection. Physicians should be aware of this diagnosis while managing nonhealing skin lesions in patients with chronic lymphedema and vascular insufficiency. A delay in the diagnosis of angiosarcoma could culminate in significant treatment challenges. Radiotherapy alone may be an effective treatment in a select group of patients with cutaneous angiosarcoma of the foot in cases where surgery is not feasible. An excellent subjective and objective response to radiation was achieved in our case.

## References

[1] Mark R. J., Poen J. C., Tran L. M., Fu Y. S., Juillard G. F. (1996). Angiosarcoma: a report of 67 patients and a review of the literature. *Cancer*.

[2] Weiss S. W., Goldblum J. R., Folpe A. L. (2007). *Enzinger and Weiss's Soft Tissue Tumors*.

[3] Penel N., Lansiaux A., Adenis A. (2007). Angiosarcomas and taxanes. *Current Treatment Options in Oncology*.

[4] Trinh N. Q., Rashed I., Hutchens K. A., Go A., Meilan E., Tung R. (2013). Unusual clinical presentation of cutaneous angiosarcoma masquerading as eczema: a case report and review of the literature. *Case Reports in Dermatological Medicine*.

[5] Holden C. A., Spittle M. F., Jones E. W. (1987). Angiosarcoma of the face and scalp, prognosis and treatment. *Cancer*.

[6] Nagao K., Suzuki K., Yasuda T. (2013). Cutaneous angiosarcoma of the buttock complicated by severe thrombocytopenia: a case report. *Molecular Clinical Oncology*.

[7] Wolf K., Pasquino J. (1990). Cutaneous angiosarcoma. A literature review and case report. *Journal of the American Podiatric Medical Association*.

[8] Selim A., Khachemoune A., Lockshin N. A. (2005). Angiosarcoma: A case report and review of the literature. *Cutis*.

[9] Young R. J., Brown N. J., Reed M. W., Hughes D., Woll P. J. (2010). Angiosarcoma. *The Lancet Oncology*.

[10] Mentzel T., Kutzner H., Wollina U. (1998). Cutaneous angiosarcoma of the face: clinicopathologic and immunohistochemical study of a case resembling rosacea clinically. *Journal of the American Academy of Dermatology*.

[11] Shehan J. M., Ahmed I. (2006). Angiosarcoma arising in a lymphedematous abdominal pannus with histologic features reminiscent of Kaposi's sarcoma: report of a case and review of the literature. *International Journal of Dermatology*.

[12] Knight T. E., Robinson H. M., Sina B. (1980). Angiosarcoma (angioendothelioma) of the scalp. An unusual case of scarring alopecia. *Archives of Dermatology*.

[13] Pan Z., Albertson D., Bhuller A., Wang B., Shehan J. M., Sarma D. P. (2008). Angiosarcoma of the scalp mimicking a sebaceous cyst. *Dermatology Online Journal*.

[14] McCarthy W., Pack G. (1950). Malignant blood vessel tumor. *Surgery Gynecology Obstetrics*.

[15] Morgan M. B., Swann M., Somach S., Eng W., Smoller B. (2004). Cutaneous angiosarcoma: a case series with prognostic correlation. *Journal of the American Academy of Dermatology*.

[16] Ogawa K., Takahashi K., Asato Y., Yamamoto Y., Taira K., Matori S., Iraha S., Yagi N., Yogi A., Haranaga S., Fujita J., Uezato H., Murayama S. (2012). Treatment and prognosis of angiosarcoma of the scalp and face: A retrospective analysis of 48 patients. *The British Journal of Radiology*.

[17] Freedman A. N. (1987). Angiosarcoma of the scalp: case report and literature review. *Canadian Journal of Surgery*.

[18] Girard C., Johnson W. C., Graham J. H. (1970). Cutaneous angiosarcoma. *Cancer*.

[19] Balaji G., Arockiaraj J. S. V., Roy A. C., Deepak B. (2014). Primary epithelioid angiosarcoma of the calcaneum: a diagnostic dilemma. *Journal of Foot and Ankle Surgery*.

[20] Choi S. Y., Min S. K., Kim K. I., Kim H. Y. (2012). Intimal angiosarcoma presenting with common femoral artery aneurysm. *Journal of Vascular Surgery*.

[21] Köhler H. F., Neves R. I., Brechtbühl E. R., Granja N. V. M., Ikeda M. K., Kowalski L. P. (2008). Cutaneous angiosarcoma of the head and neck: report of 23 cases from a single institution. *Otolaryngology—Head and Neck Surgery*.

[22] Kacker A., Antonescu C. R., Shaha A. R. (1999). Multifocal angiosarcoma of the scalp: a case report and review of the literature. *Ear, Nose & Throat Journal*.

[23] Lydiatt W. M., Shaha A. R., Shah J. P. (1994). Angiosarcoma of the head and neck. *The American Journal of Surgery*.

[24] Guadagnolo B. A., Zagars G. K., Araujo D., Ravi V., Shellenberger T. D., Sturgis E. M. (2011). Outcomes after definitive treatment for cutaneous angiosarcoma of the face and scalp. *Head and Neck*.

[25] Naka N., Ohsawa M., Tomita Y. (1998). Prognostic factors in angiosarcoma: a multivariate analysis of 55 cases. *Journal of Surgical Oncology*.

[26] Maddox J. C., Evans H. L. (1981). Angiosarcoma of skin and soft tissue: a study of forty-four cases. *Cancer*.

[27] Kharkar V., Jadhav P., Thakkar V., Mahajan S., Khopkar U. (2012). Primary cutaneous angiosarcoma of the nose. *Indian Journal of Dermatology, Venereology and Leprology*.

[28] Pawlik T. M., Paulino A. F., McGinn C. J. (2003). Cutaneous angiosarcoma of the scalp: a multidisciplinary approach. *Cancer*.

[29] Kitagawa M., Tanaka I., Takemura T., Matsubara O., Kasuga T. (1987). Angiosarcoma of the scalp: report of two cases with fatal pulmonary complications and a review of Japanese autopsy registry data. *Virchows Archiv A*.

[30] Nomura M., Nakaya Y., Saito K., Miyoshi H., Kishi F., Hibino S., Saijyo T., Ito S., Nakagawa K., Nakanishi H., Nagae H., Toda N., Tanaka S., Harada H., Matsumoto K., Hasegawa T. (1994). Hemopneumothorax secondary to multiple cavitary metastasis in angiosarcoma of the scalp. *Respiration*.

[31] Mendenhall W. M., Mendenhall C. M., Werning J. W., Reith J. D., Mendenhall N. P. (2006). Cutaneous angiosarcoma. *The American Journal of Clinical Oncology: Cancer Clinical Trials*.

[32] Ward J. R., Feigenberg S. J., Mendenhall N. P., Marcus R. B., Mendenhall W. M. (2003). Radiation therapy for angiosarcoma. *Head and Neck*.

[33] Morrison W. H., Byers R. M., Garden A. S., Evans H. L., Ang K. K., Peters L. J. (1995). Cutaneous angiosarcoma of the head and neck. Atherapeutic dilemma. *Cancer*.

[34] Glickstein J., Sebelik M. E., Lu Q. (2006). Cutaneous angiosarcoma of the head and neck: a case presentation and review of the literature. *Ear, Nose & Throat Journal*.

[35] Romanyshyn J., Wolden S., Caria N. (2010). Radiation therapy in the treatment of angiosarcoma of the head and neck. *International Journal of Radiation Oncology, Biology, Physics*.

[36] Gkalpakiotis S., Arenberger P., Vohradnikova O., Arenbergerova M. (2008). Successful radiotherapy of facial angiosarcoma. *International Journal of Dermatology*.

[37] Patel V. B., Speer T. W. (2012). Successful treatment of an angiosarcoma of the nose with radiation therapy. *Case Reports in Oncology*.

[38] Owens J. C., Shiu M. H., Smith R., Hajdu S. I. (1985). Soft tissue sarcomas of the hand and foot. *Cancer*.

[39] Simon M. A., Enneking W. F. (1976). The management of soft tissue sarcomas of the extremities. *Journal of Bone and Joint Surgery A*.

[40] Penel N., Bui B. N., Bay J.-O. (2008). Phase II trial of weekly paclitaxel for unresectable angiosarcoma: the ANGIOTAX study. *Journal of Clinical Oncology*.

[41] Agulnik M., Okuno S., Von Mehren M. (2009). An open-label multicenter phase II study of bevacizumab for the treatment of angiosarcoma. *Journal of Clinical Oncology*.

[42] George S., Merriam P., Maki R. G., Van Den Abbeele A. D., Yap J. T., Akhurst T., Harmon D. C., Bhuchar G., O'Mara M. M., D'Adamo D. R., Morgan J., Schwartz G. K., Wagner A. J., Butrynski J. E., Demetri G. D., Keohan M. L. (2009). Multicenter phase II trial of sunitinib in the treatment of nongastrointestinal stromal tumor sarcomas. *Journal of Clinical Oncology*.

[43] Maki R. G., D'Adamo D. R., Keohan M. L., Saulle M., Schuetze S. M., Undevia S. D., Livingston M. B., Cooney M. M., Hensley M. L., Mita M. M., Takimoto C. H., Kraft A. S., Elias A. D., Brockstein B., Blachère N. E., Edgar M. A., Schwartz L. H., Qin L.-X., Antonescu C. R., Schwartz G. K. (2009). Phase II study of sorafenib in patients with metastatic or recurrent sarcomas. *Journal of Clinical Oncology*.

[44] Thong P. S.-P., Ong K.-W., Goh N. S.-G., Kho K.-W., Manivasager V., Bhuvaneswari R., Olivo M., Soo K.-C. (2007). Photodynamic-therapy-activated immune response against distant untreated tumours in recurrent angiosarcoma. *The Lancet Oncology*.

